# Can weight status in the first year of life predict well-being in the first years of school?

**DOI:** 10.1038/s41366-026-02180-6

**Published:** 2026-08-20

**Authors:** Mie Sylow Liljendahl, Mette Rasmussen, Sofie Weber Pant, Lis Marie Pommerencke, Lau Caspar Thygesen, Trine Pagh Pedersen

**Affiliations:** National Institute of Public Health, University of Southern Denmark, Copenhagen, Denmark

**Keywords:** Epidemiology, Risk factors

## Abstract

**Background:**

Early life represents a critical period for shaping long-term health outcomes, with early interventions playing a significant role in promoting well-being. This study aimed to investigate whether infant weight status could predict concerns of development and well-being in the first years of school using health nurse data.

**Methods:**

Data from the Danish Child Health Database collaboration included 54 917 children and families who received home visits at 8–10 months and school examinations by health nurses during the children’s first years of school. Weight status was measured using BMI z-scores and tested categorical, linear and using restricted cubic splines to explore predictive performance. Well-being was assessed by health nurses using seven indicators, including language skills, motor development, emotional well-being, and peer relationship problems. Model performance was evaluated through visualization of restricted cubic splines and by Area Under the Curve (AUC).

**Results:**

Infant weight status at 8–10 months showed poor predictive performance (AUC < = 0.533) across all indicators of well-being in the first years of school. While certain weight categories—especially severe thinness, overweight, and obesity—were linked to specific concerns such as language skills and motor development, these associations had wide confidence intervals.

**Conclusion:**

Although infant weight status may indicate later health concerns, it was not a reliable predictor of health nurses’ well-being concerns in the early school years. The findings demonstrated that predictive performance remained poor across several models, suggesting that infant weight alone may not capture the complexity of factors influencing later well-being as recorded by health nurses.

## Background

Early life represents a critical period for shaping long-term trajectories of well-being [1]. Consequently, early interventions play a key role in fostering healthy life courses from childhood through adolescence into adulthood [1, 2]. In certain instances, early interventions—such as selective health promotion initiatives—are enhanced by or may require early identification of children at risk of adverse outcomes. Health nurses may identify these children, and from both cost-efficiency and ethical perspectives, it is essential to evaluate the potential of routinely collected data within the child healthcare system.

Two major health challenges that manifest early in childhood and impact daily lives are poor well-being [3, 4] and excessive weight [5–7]. The literature further documents significant associations between weight status and various indicators of well-being among children and adolescents. Specifically, overweight and obesity have been linked to reduced quality of life [8–13], low self-esteem, feelings of sadness, loneliness, nervousness, and depression [14–17]. Additionally, excessive weight is associated with lower socioeconomic status, reduced social participation [18], and experiences of bullying [19–21]. While the relationship may be bidirectional [22, 23], prospective longitudinal studies suggest that childhood obesity increases the risk of subsequent depression, low self-esteem, and body dissatisfaction [14, 17, 24, 25]. Emerging evidence also suggests that excessive weight in childhood may adversely affect developmental outcomes. Studies have reported that obesity may precede declines in motor skills, indicating that early obesity prevention could help maintain motor skills and may positively impact physical activity and overall fitness levels [26]. Similarly, higher adiposity has been associated with poorer gross motor coordination and neuromuscular performance among children and adolescents [27]. Evidence regarding language development is less consistent. A systematic review found mixed results, with some studies indicating language difficulties or lower reading skills among children with obesity, while others reported no clear associations, particularly in younger children [28]. Together, these findings indicate that the consequences of childhood overweight and obesity may extend beyond physical and psychosocial health to broader aspects of child development.

Given the challenges associated with poor well-being among children in Western societies, a range of initiatives and structural interventions are being implemented and evaluated to enhance child well-being [29–31]. A key component in establishing selective and indicated preventive interventions is identifying early-life indicators capable of predicting poor well-being in childhood. In light of existing evidence linking weight status to well-being, early-life weight status emerges as a potential predictive marker. However, the literature has predominantly focused on prediction of weight status, particularly the prediction of overweight and obesity in children and adolescents [32]. Studies specifically examining whether weight status predicts health outcomes have primarily focused on subsequent disease risk [33, 34]. Consequently, this leaves a gap regarding whether weight status in infancy can serve as a predictor of well-being in the early school years.

In addition to its role in clinical work and services, it is worth investigating whether record data on infant weight status routinely collected by Danish health nurses may have predictive value for subsequent child well-being. Establishing such predictive value could lay the groundwork for targeted health-promoting initiatives aimed at improving childhood well-being. Therefore, the objective of this study was to examine whether infant weight status in the first year of life can predict concerns of development and well-being in the early school years, based on health nurse assessments.

## Method

### Study design, population, and setting

In Denmark, health nurses are integrated into all municipal healthcare systems, providing comprehensive child health promotive services, including free, scheduled home visits for all families with a newborn. In alignment with recommendations from the Danish Health Authority, families are offered a minimum of five home visits during the first postnatal year [35]. Although participation in the home visiting program is voluntary, it is widely utilized, with 98–99% of families in Denmark receiving regular home visits during their child’s first year of life [36]. Health nurses systematically document child health records, which contain detailed information on developmental milestones, including weight status and growth trajectories in infancy. Municipalities in Denmark are legally required to offer children and their families school examinations during the first and final school years, provided by health nurses [35]. Attendance during the first school years is near-universal [37].

This study is based on data from the Danish Child Health Database collaboration [38–42]. This database includes data from standardized recordings at home visits during infancy and school examinations, collected in 33 out of 98 municipalities in Denmark. From 2002 until 2023, the number of included municipalities has varied. The Danish Child Health Database includes records of infant home visits dating back to 2002, as well as health nurse school examinations from 2007 onward. Data from the first year of life, as well as the first years of schooling, are based on examinations and observations covering a wide range of family factors and child development, supplemented by information from parental conversations, leading to an overall assessment of the child’s development, health, and well-being. All information is collected by health nurses and registered according to a standardized manual, ensuring comparability and enhancing the validity of the records [43]. The examination takes place in either kindergarten (school entry year) or first grade, usually with at least one parent present. The health nurse’s records and observations on the child’s development and well-being are based on a conversation with the child and parents and a physical examination. In addition to the health nurses’ observations, parents are asked to complete a questionnaire that provides additional insights into the child’s health, medical history, and social interactions prior to the examination.

The current study was designed as a prospective cohort of children followed from a health nurse visit between ~8–10 months after birth (age range: 7.3–10.7 months) to their first school examination by a health nurse between 6 and 7 years of age (age range: 5.2–9.4 years). The database comprised 302,539 children with records from the first year of life and 113,047 at first years of school. Inclusion in the study required available data at both time points. The study population (*n* = 69,161) consisted of children who received home visits and underwent a school health nurse examination during their early school years between 2007 and 2023. Children with missing or unrealistic information on weight or recumbent length were excluded, resulting in a final study population of 54,917 children. A flowchart illustrating the participant inclusion and exclusion process is provided in Fig. S1. Most of the children were born between 2013 and 2018 (54.2%).

### Predictor variable

The predictor variable—infant weight status—was based on the measurements of weight and recumbent length at age 8–10 months, assessed by the health nurse. The Danish Health Authority recommends that infants be weighed without diaper and wearing only light clothing, using a handheld hanging scale. Recumbent length should be measured with the child in a supine position on a firm surface, preferably using a length board [37]. According to official recommendations, all personnel performing length measurements should receive adequate training, and measurements should ideally be conducted by two individuals. One person positioned the child’s head against the fixed headpiece, while the other extended the legs and placed the feet against the movable footboard. The Danish Health Authority recommends the weight be measured in kilograms, rounded to one decimal place, and that recumbent length be measured to the nearest half centimeter [37].

Weight status was determined using the WHO definition of BMI z-scores for children aged 8–10 months [44]. If multiple measurements were available for the same child during the interval of 8–10 months, the most recent measurement was used to classify weight status. The calculation of BMI z-scores included age, weight, and length. If any of these three variables were missing, the weight status was recorded as missing. For children with missing information, supplementary information on age, weight, and length at 8–10 months was retrieved from the National Child Database [45]. The National Child Database contains health nurse data about height, weight, passive smoking, and breastfeeding from all municipalities in Denmark [45]. The proportion of missing values for BMI z-score was 20.6%. Further, weight status was categorized as severe thinness (BMI z-score <-3), thinness (BMI z-score >=-3 & <-2), normal weight (BMI z-score >=-2 & <=1), risk of overweight (BMI z-score >1 & <=2), overweight (BMI z-score >2 & <=3) and obesity (BMI z-score >3) in accordance with WHO’s classification for children up to 5 years of age [46]. In addition to these categories, BMI z-scores were also analyzed as a continuous variable.

Few children had very low and very high BMI z-scores. To ensure anonymization of data, the scale of BMI z-scores was therefore aggregated into nearest integer between −4 and 5. Children with higher or lower estimates of BMI z-score were included in the highest (5) or lowest level (-4).

### Outcomes

The outcomes of development and well-being included in this study were based on available data from The Danish Child Health Database and are interpreted as component measures or indicators of child development and well-being. Development and well-being were defined by any concerns reported in seven different development and well-being measurements by the health nurses at the examination in the first years of school. These measurements included: language skills, motor development, the child’s perceived general well-being, as well as perceived school well-being, parent-child relationship, health nurses’ observations of the interaction between the child and the health nurse, and parent-reported perspectives on peer relationship problems. If a child does not develop age-appropriately, health nurses record a concern indicating that something is unusual or requires attention.

The outcomes of this study were categorized as binary variables (yes/no) and defined as follows:

Children’s language skills were recorded as either a concern or no concern. Between 2007 and 2017, a concern was noted if the child demonstrated pronunciation errors, stuttering, age-inappropriate vocabulary, or did not ask questions. From 2018 onwards, the criteria were expanded to include the child shows pronunciation difficulties, gives limited or irrelevant answers, struggles to maintain a topic or tell a coherent story, rarely asks questions, has challenges with attention and concentration, or is unable to follow instructions consisting of at least two steps. A concern may also be noted if issues related to stuttering or limited language use are observed (the changes had no impact on the frequency of concerns).

Motor developments were assessed through observations in several activities. A child was classified as having a concern if they exhibited two or more difficulties in the following activities: walking, jumping, coordinated running, standing on one leg (with right and left legs combined into one category), throwing a ball (with right and left hands combined into one category), preferred hand usage, and grip strength.

General and school well-being were self-assessed by the child using four smiley-face options: “Very happy,” “Happy,” “In between,” and “Not happy.” A concern was recorded if the child answered “In between” or “Not happy” when asked “How do you usually feel?” or “How do you usually feel at school?”.

Observations related to the parent–child relationship included the documentation of concerns, if present. Between 2007 and 2018, such concerns were noted if the child appeared unkempt or inadequately dressed for the weather or season; if the parents failed to respond to the child’s cues; or if other observations indicated potential neglect. From 2018 onwards, the criteria were revised to encompass whether the child seeks support, whether the parents provide motivation and encouragement, and whether there is evidence of mutual engagement. Additionally, a concern may also address the quality of physical caregiving and other facets of parent–child contact (the changes had no impact on the frequency of concerns).

Observations and interactions between the health nurse and the child were assessed, and concerns were noted if the child appeared sad; was so shy that it limited their ability to engage in activities; displayed restlessness or lack of concentration; was consistently argumentative; avoided eye contact; exhibited boundary-testing behavior; showed rejection or withdrawal; was unable to follow instructions; or demonstrated other noteworthy behaviors related to emotional state.

The questions on peer relationships were derived from the Danish version of the internationally validated Strengths and Difficulties Questionnaire (SDQ) [47] and constitute the “Peer Relationship Problems” subscale [48], which is comparable to the British version [49]. As part of the school examination, parents are asked to assess their child’s peer relationships using a questionnaire completed before the examination. The health nurse reviews the responses in dialogue with the parents and child. The subscale consists of several parent-reported observations combined into a single outcome, including “Rather solitary, tends to play alone,” “Has at least one good friend,” “Generally liked by other children,” “Picked on or bullied by other children,” and “Gets on better with adults than with other children.” Parents rate each statement using three response categories: *“Not true,”* “*Somewhat true,”* and *“Certainly true.”* Responses are summed to a total score ranging from 0 to 10, with higher scores indicating more difficulties. A minimum of three responses is required for inclusion in the final score [49]. Scores were classified according to Danish norms as follows: 0–2 (normal range), indicating no problems, and 3–10 (borderline and outside the normal range), indicating signs of problems [50].

### Statistics

The correlation between the seven development and well-being outcomes was assessed using tetrachoric correlation for descriptive purposes, to measure agreement rates and the extent to which the questions capture distinct aspects. To predict development and well-being in the first years of school, we constructed a logistic regression model to analyze the association between weight status at 8–10 months and development and well-being outcomes in the first years of school. Categorized weight status was tested as a predictor separately for all previously mentioned development and well-being outcomes. We assessed each weight category’s contribution to predictive performance to identify categories with greater influence on accuracy. Additionally, weight status was analyzed using BMI z-score as a linear predictor for all seven development and well-being indicators and, finally, using restricted cubic splines to explore potential non-linear relationships between BMI z-score and all indicators. The knots for the restricted cubic splines regression were placed at (−1, 0, 1) based on 25, 50 and 75th percentiles.

To assess the predictive performance, all models were evaluated by calculating Area Under the Curve (AUC (c-statistics)) and restricted cubic splines were presented through visualization.

Data processing and analyses were conducted using R statistical software version 4.1.0 (2021-5-18).

### Sensitivity and sub-analyses

For sensitivity, all models were tested using a simplified version of the WHO’s BMI-for-age classification divided into categories: severe thinness/thinness, normal weight, risk of overweight, and overweight/obesity. To address methodological challenges related to the use of BMI-for-age, we conducted a sensitivity analysis using weight-for-length as well. Furthermore, we conducted two separate sub-analyses excluding children with concerns about well-being in the age of 8–10 months. In the first analysis, we excluded children whose health nurse reported at least two concerns, such as motor development, communication, sleep, crying, eating, parent-infant relation, or parental mental condition. In the second analysis, we excluded children with three or more reported concerns. These sensitivity analyses assumed that children showing signs of well-being concerns at 8–10 months would also be identified as having concerns about well-being during their first years of school, thereby serving as an early underlying predictor. Accordingly, concerns about well-being were examined as a co-predictor of weight status to test this.

As sub-analysis, all variables used to define the child’s peer relationship problems were tested as individual outcomes. Further sub-analyses included stratification of sex (girl and boy) and stratification on educational level (low and high level of education). Using the unique personal identification number (CPR number) assigned to all citizens in Denmark, infants were linked to parental education data from the Population Educational Register [51]. Classification was based on the highest level of education completed by either parent at the child’s year of birth, ranging from primary and lower secondary education (low education level) to three or more years at university (high education level). If educational data were available for only one parent, classification was based on that parent’s education.

## Results

Half of the population were girls (49.5%), 39.0% had parents with a low level of education, and 4.0% had parents who were unemployed. The majority of parents were of Danish ethnic origin (73.3%); 41.7% of the children were firstborns; and most were born to term (94.2%). Regarding weight status in infancy, 0.2% of children were classified with severe thinness; 2.0% with thinness; 77.2% with normal weight; 16.4% at risk of overweight; 3.7% with overweight; and 0.5% with obesity (Table 1). According to health nurses’ assessments during health examinations in the first years of school, concerns about children’s development and well-being were distributed as follows: 9.8% of children had concerns related to language skills, 8.6% to motor development, 5.1% to the child’s perceived general well-being, 10.6% to the child’s perceived school well-being, 5.0% to the parent-child relationship, 14.5% based on health nurse-observed child interaction, and 7.4% regarding peer relationship problems. Overall, 63.4% of the children had no concerns noted in the health nurses’ reports, 23.3% had one concern, 8.7% had two concerns, and 4.6% had three or more concerns.

**Table 1:** Baseline characteristics of the study population.

	N (%)
Girls	27,211 (49.5)
Boys	27,706 (50.5)
**Firstborns**	
Yes	22,873 (41.7)
No	32,044 (52.3)
**Born to term**	
Yes	50,725 (94.2)
No	3123 (5.8)
**Weight status in infancy**	
Severe thinness	103 (0.2)
Thinness	1112 (2.0)
Normal weight	42,377 (77.2)
Risk of overweight	9004 (16.4)
Overweight	2045 (3.7)
Obesity	276 (0.5)
**Parental education level**	
Low level of education	21,314 (39.0)
High level of education	33,383 (61.0)
**Parental employment status**	
Unemployed	2186 (4.0)
Employed	52,165 (96.0)
**Parental ethnic origin**	
Both parents of Danish origin	40,280 (73.3)
One Danish parent and one immigrant or descendant	5857 (10.7)
Both parents immigrants or descendants	8778 (16.0)

First-born status and born to term were obtained from the Danish Medical Birth Register; Parental education was obtained from the Population Education Register; parental employment from the Employment Classification Module; parental origin from the Danish Civil Registration System.

The correlation assessed using tetrachoric correlation demonstrated no strong correlation between development and well-being outcomes in the first years of school. The strongest correlation was observed between perceived general well-being and perceived school well-being, with a correlation coefficient of 0.505. Similarly, only moderate correlation was found between the measurements used for peer relationship problems. The strongest correlation was between the “Rather solitary, tends to play alone” and “Gets on better with adults than with other children” with a correlation coefficient of 0.590 (data not shown).

We evaluated the predictive performance of a logistic regression model for concerns about development and well-being by weight status in infancy using AUC, which demonstrated low performance ( ≤ 0.51) across all outcomes. When assessing AUC by weight status category, we found that the severe thinness, overweight and obesity categories predominantly influenced the AUC for several outcomes (Table 2). The odds ratio revealed higher odds for concerns about development and well-being outcomes in certain weight status categories (Table 3). Severe thinness and thinness in infancy were associated with higher odds of concerns regarding language skills in childhood (OR 1.82, 95% CI:1.08–2.91; OR 1.24, 95% CI:1.04–1.47). Children with overweight in infancy had higher odds of concerns regarding motor development (OR 1.22, 95% CI:1.07–1.39), the child’s perceived school well-being (OR 1.25, 95% CI: 1.10–1.40), health nurse-observed child interaction (OR 1.14, 95% CI:1.02–1.28), and peer relationship problems (OR 1.29, 95% CI: 1.11–1.48). Conversely, children with obesity in infancy had higher odds of concerns related to language skills (OR 1.47, 95% CI:1.05–2.02) (Table 3). The odds ratios stratified by sex and parental level of education were similar to the previously reported estimates (data not shown).

**Table 2:** Performance of logistic-, linear- and restricted cubic spline regression measured in Area Under the Curve (AUC) and performance by stratification of weight status for logistic regression.

	Concerns about language skills	Concerns about motor development	Concerns about perceived general well-being	Concerns about perceived school well-being	Concerns about parent-child relationship	Concerns based on health nurse observations on child interaction	Concerns about peer relationship problems
**Overall AUC**							
Weight status, categorical	0.507	0.508	0.507	0.510	0.504	0.504	0.509
BMI z-score, linear	0.501	0.503	0.510	0.507	0.503	0.501	0.505
BMI z-score, restricted cubic splines	0.514	0.517	0.513	0.511	0.512	0.509	0.513
**Individual AUC for weight status by logistic regression**							
Severe thinness	0.533	0.521	0.503	0.517	0.502	0.504	0.502
Thinness	0.510	0.506	0.502	0.505	0.501	0.503	0.504
Normal weight	0.503	0.503	0.502	0.505	0.501	0.501	0.503
Risk of overweight	0.500	0.500	0.501	0.502	0.501	0.501	0.501
Overweight	0.505	0.508	0.503	0.511	0.500	0.509	0.510
Obesity	0.519	0.510	0.502	0.508	0.503	0.501	0.507

**Table 3:** Proportion and odds ratio for concerns of development and well-being in first years of school by weight status in first year of life (8–10 months).

	% concerns	OR (95%CI)
Concerns about language skills		
Severe thinness	16.2	1.82 (1.08–2.91)
Thinness	11.7	1.24 (1.04–1.47)
Normal weight	9.6	1
Risk of overweight	9.7	1.01 (0.94–1.09)
Obesity	13.6	1.47 (1.05–2.02)
Concerns about motor development		
Severe thinness	12.4	1.42 (0.84–2.26)
Thinness	9.5	1.17 (0.98–1.40)
Normal weight	8.2	1
Risk of overweight	8.3	1.02 (0.95–1.09)
Overweight	9.8	1.22 (1.07–1.39)
Obesity	10.4	1.28 (0.91–1.80)
Concerns about perceived general well-being		
Severe thinness	4.7	0.91 (0.36–1.90)
Thinness	5.5	1.08 (0.85–1.35)
Normal weight	5.2	1
Risk of overweight	5.5	1.07 (0.97–1.16)
Overweight	5.9	1.15 (0.97–1.35)
Obesity	5.7	1.11 (0.68–1.69)
Concerns about perceived school well-being		
Severe thinness	14.2	1.44 (0.83–2.26)
Thinness	11.4	1.13 (0.95–1.32)
Normal weight	10.5	1
Risk of overweight	11.1	1.06 (1.00–1.14)
Overweight	12.7	1.25 (1.10–1.40)
Obesity	12.2	1.17 (0.85–1.61)
Concerns about parent-child relationship		
Severe thinness	4.2	0.91 (0.32–2.01)
Thinness	4.6	0.98 (0.74–1.26)
Normal weight	4.6	1
Risk of overweight	4.9	1.05 (0.96–1.16)
Overweight	4.6	0.97 (0.82–1.21)
Obesity	4.1	0.87 (0.47–1.45)
Concerns based on health nurses’ observations on child interaction		
Severe thinness	13.2	0.94 (0.54–1.54)
Thinness	14.5	1.05 (0.90–1.22)
Normal weight	14.0	1
Risk of overweight	13.8	0.99 (0.93–1.05)
Overweight	15.7	1.14 (1.02–1.28)
Obesity	13.8	0.99 (0.72–1.33)
Concerns about peer relationship problems		
Severe thinness	7.6	1.07 (0.50–1.99)
Thinness	8.0	1.13 (0.92–1.38)
Normal weight	7.1	1
Risk of overweight	7.4	1.04 (0.96–1.13)
Overweight	9.0	1.29 (1.11–1.48)
Obesity	5.9	0.83 (0.50–1.27)

Percentages represent the proportion of children within each weight category who were assessed by the health nurse as having the specific concern.

Given the limited number of observations in the outer categories of the WHO’s BMI-for-age classification, all models were re-tested using a simplified classification. Results from these simplified categories did not show changes compared to the original classification, with similar performance in AUC (data not shown).

**Fig. 1:**
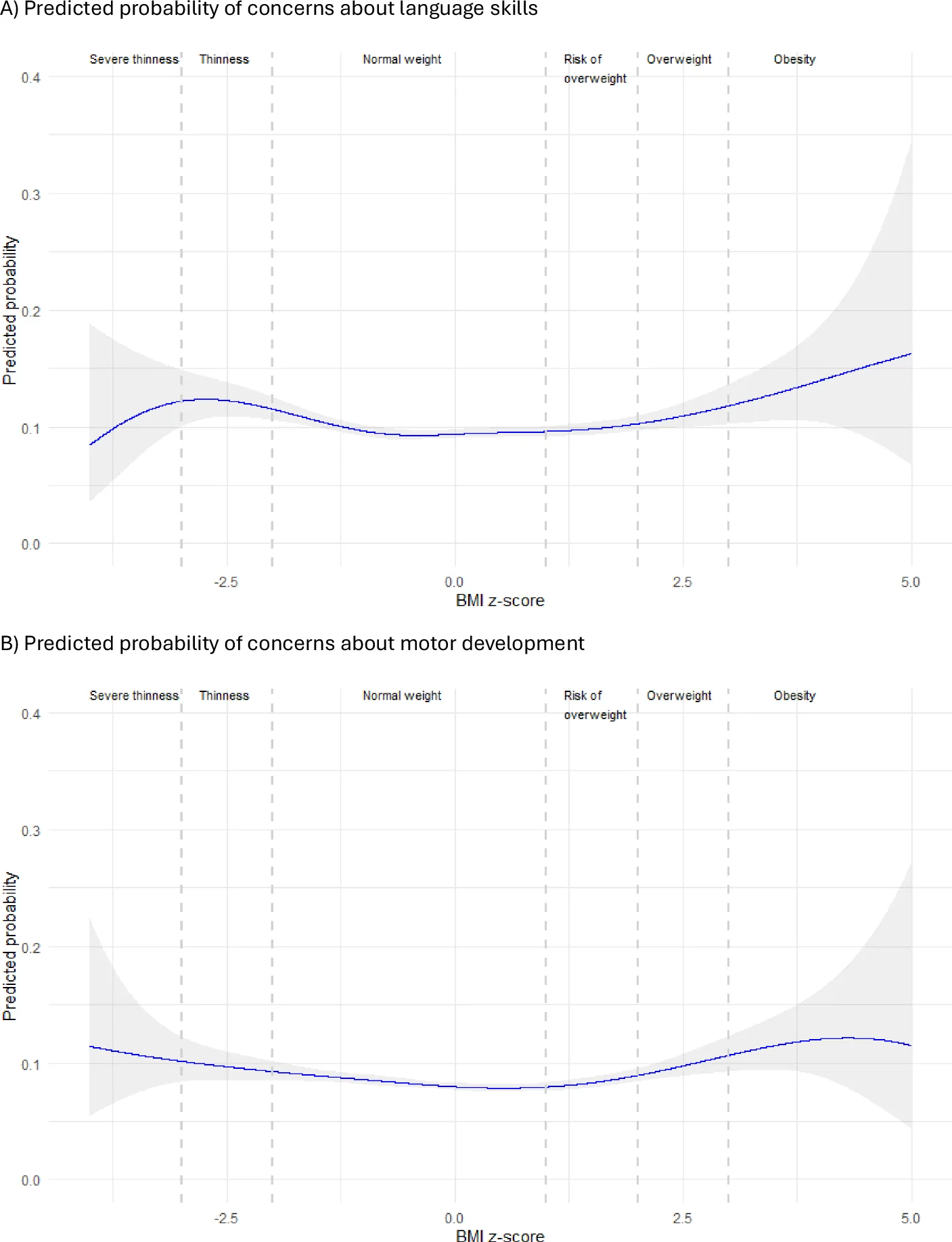
Predicted probability of concerns about the child’s development and well-being during the first years of school according to BMI z-score at 8–10 months. **A**, **B** Predicted probability and 95% confidence intervals for concerns about language skills and motor development in first years of school by BMI z-score at 8–10 months.

**Fig. 2:**
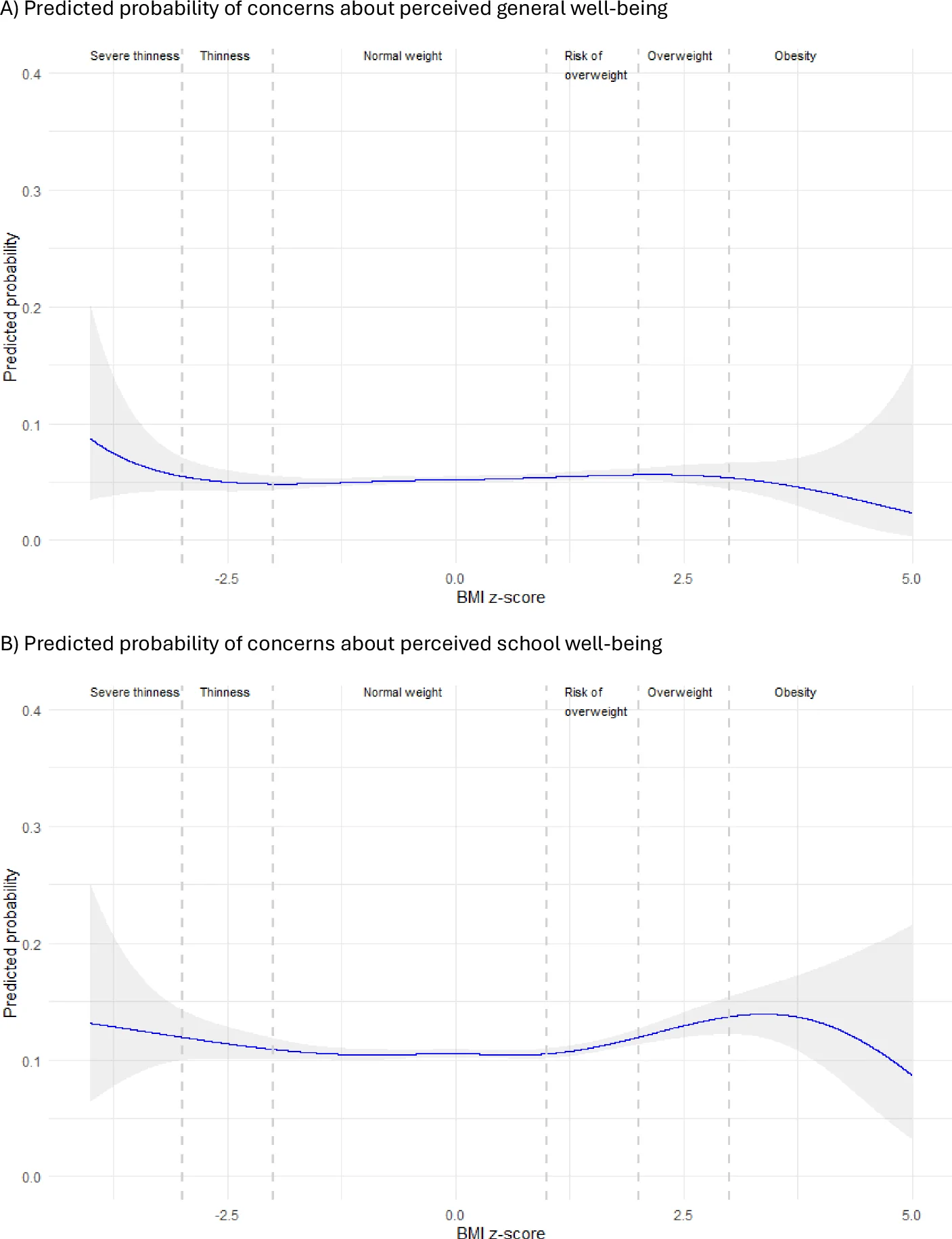
Predicted probability of concerns about the child’s development and well-being during the first years of school according to BMI z-score at 8–10 months. **A**, **B** Predicted probability and 95% confidence intervals for concerns about the child’s perceived general well-being and perceived school well-being in first years of school by BMI z-score at 8–10 months.

**Fig. 3:**
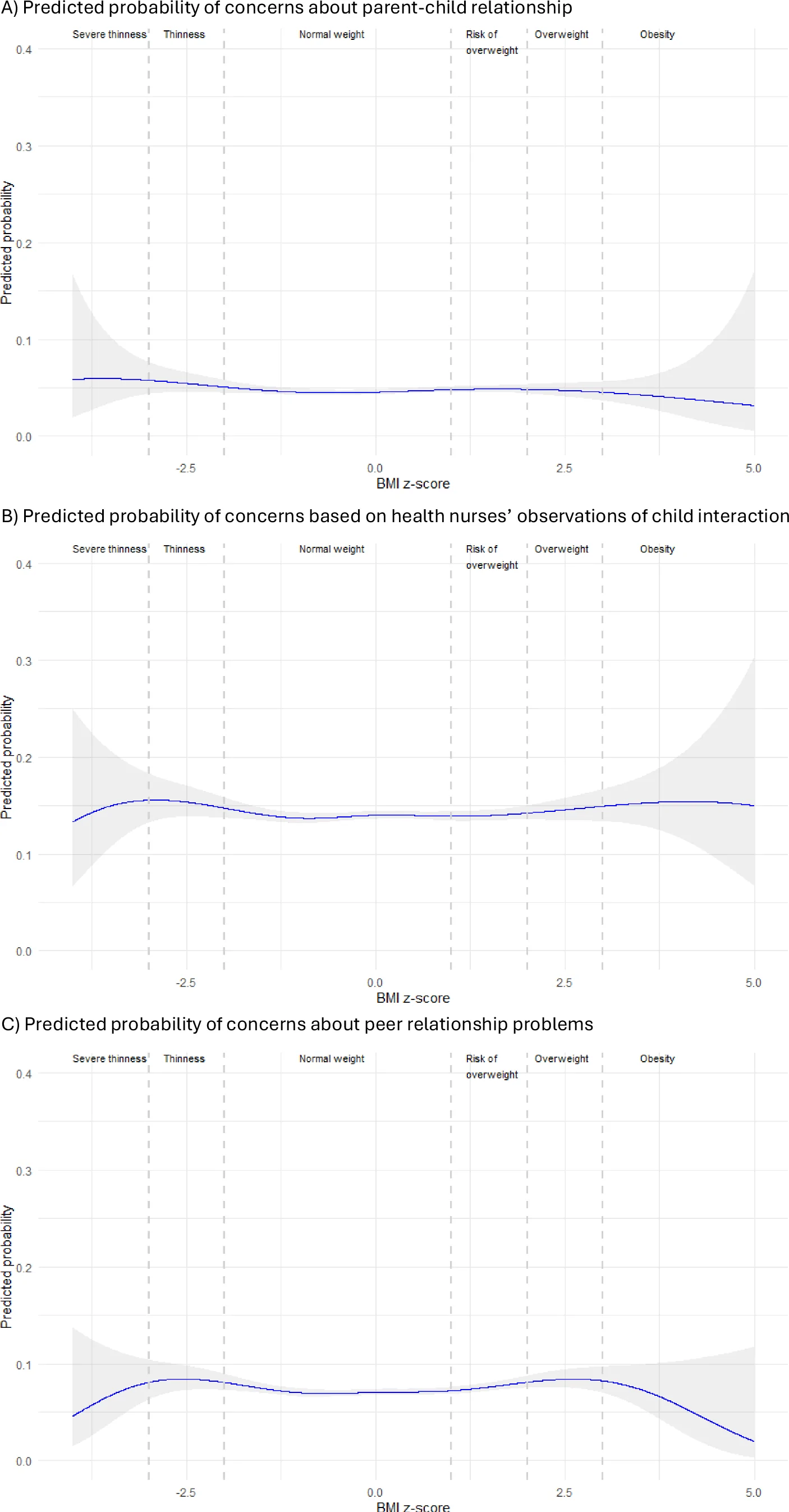
Predicted probability of concerns about the child’s development and well-being during the first years of school according to BMI z-score at 8–10 months. **A**–**C** Predicted probability and 95% confidence intervals for concerns about parent-child relationship, health nurses’ observations on child interaction and peer relationship problems in first years of school by BMI z-score at 8–10 months.

No linear relationship was found between BMI z-scores and development and well-being outcomes. The predictive performance demonstrated an AUC ranging from 0.50 to 0.51 (Table 2). Using a cubic spline, the highest AUC observed for concerns about language skills and motor development demonstrated an AUC of 0.514 and 0.517, with slightly lower AUCs observed for other outcomes. The restricted cubic splines performed slightly better than those using weight status as a categorical or continuous variable, but still exhibited poor predictive performance (Table 2). The predicted probability with corresponding 95% confidence intervals for concerns about development and well-being by BMI z-score is presented as spline curves in Figs. 1–3. No substantial improvement in the predictive performance was observed for weight-for-length (data not shown).

Excluding children with three or more concerns indicating poor well-being at 8–10 months (*n* = 3 758) did not improve the performance of AUC when using restricted cubic spline regression. Similarly, when excluding children with two or more concerns during 8–10 months (*n* = 7 012), the AUC was not improved. Accordingly, concerns about well-being were examined as a co-predictor of weight status; however, it did not significantly improve the AUC (data not shown). Similarly, the predictive performance of the individual items of concern related to the child’s peer relationship problems yielded poor AUCs. The predictive performance of using restricted cubic splines stratified by sex showed no differences in AUC for girls compared to boys. The overall AUC remained low, indicating poor predictive ability. Similarly, no clear pattern in AUC was observed when stratified by high and low education level (Table S1).

## Discussion

This study examined the predictive performance of infant weight status, recorded as part of routinely collected data by Danish health nurses during home visits at 8–10 months, in relation to various development and well-being indicators assessed at routine school examinations during the first years of school. Across all outcomes, the predictive performance of logistic regression models was poor. Similarly, when weight status was analyzed as a continuous variable using BMI-for-age z-scores, no meaningful improvement in predictive value was observed. Although models employing restricted cubic spline demonstrated slightly higher AUC values, the predictive performance remained poor. These findings suggest that infant weight at 8–10 months, whether treated categorically or continuously, is not a reliable standalone predictor of concerns of later development and well-being.

Certain weight categories—particularly severe thinness, overweight, and obesity in infancy—appeared to have a higher influence on the AUC, likely due to greater variability in health-related indicators within these groups. Further, the odds ratios indicated that children with severe thinness or thinness were more likely to elicit concerns from health nurses regarding language skills. Children with overweight had higher odds of eliciting concerns about motor development, child’s perceived school well-being, health nurses’ observations of child interaction, and peer relationship problems, whereas children with obesity showed increased odds of concerns regarding language skills. However, these associations included wide 95% confidence intervals, indicating statistical uncertainty and warranting cautious interpretation. No consistent pattern was observed when stratifying by sex and parental education level, which supports the conclusion that infant weight status alone is an insufficient predictor across subgroups.

A similar unemployment rate (3.0%) was observed in the general Danish population in 2023. The proportion of individuals of Danish ethnic origin was higher in the general population (84.6%), the proportion of citizens with a low educational level in the background population (70.3%) was markedly higher than in our study population, which may reflect both our approach of recording the highest level of education attained by either parent (when data for both was available) and the age distribution of our study population. Finally, a comparable percentage (93.6%) of children were born at term in the general population [52]. The study population was drawn from municipalities representing a mix of urban, suburban, and small towns, providing socio-demographic variation. Nevertheless, as not all regions of Denmark were included, the population may not be fully representative of the entire Danish population, and the results should therefore be interpreted in this context. In addition, the study spans several birth cohorts, and the participating families generally had a higher educational level than Danish population overall in 2023, which should also be considered when interpreting the findings.

To our knowledge, no previous studies have examined the predictive value of infant weight for later child well-being. This gap is notable, given that infant anthropometry has been widely investigated as a predictor of other outcomes, particularly later obesity. Multiple longitudinal studies have examined weight status in infancy as a predictor of childhood obesity. For example, a systematic review assessed the accuracy and feasibility of predicting overweight and obesity in children aged 3–7 years using data available in healthcare and community settings for children under 24 months [53]. The authors found that while some models incorporating longitudinal measures and machine models achieved reasonable predictive accuracy (AUCs ≥0.76), they were considered infeasible for broad-scale implementation. Simpler models, which could be more easily implemented, did not achieve sufficient accuracy for individual screening (AUCs ≥ 0.56), consistent with our results [53]. Other studies have examined early-life predictors of broader child well-being. For instance, a study applying machine learning to predict subjective well-being found that including factors such as sports participation improved predictive accuracy, highlighting specific lifestyle associations [54]. Similarly, a study using data from more than 11,000 adolescents trained neural networks to predict the risk of psychiatric disorders in adolescence [55]. The model based on current symptoms accurately identified youth who would belong to the highest-risk group in the following year (AUC = 0.84), whereas a second model relying solely on risk factors and potential disease mechanisms—but excluding current symptoms—achieved slightly lower yet still acceptable predictive accuracy (AUC = 0.75) [55]. However, a systematic review of prediction model methodology and reporting in child and adolescent mental health concluded that although many risk assessment tools have been developed recently, few are used in practice [56]. None of the new tools could currently be recommended for clinical use. Key barriers included small or unrepresentative samples and poor reporting of model performance, particularly calibration. Even models that seem to perform well should not be assumed to generalize to new populations due to methodological constraints [56].

The data originated from standardized assessments conducted by specially trained Danish health nurses who routinely interact with large numbers of children in both clinical and community settings, using a standardized assessment manual [41]. While this ensures high data quality and ecological validity, it also introduces context-specific elements that may influence assessments.

A key strength of this study is the use of the Danish Child Health Database, providing a large cohort of children with comprehensive, structured data recorded by health professionals. The database captures a wide range of development indicators and concerns related to development and well-being during routine health assessments, offering valuable insights into early childhood development and school readiness. However, the database was not originally designed for research purposes but rather as a tool for clinical support and monitoring, which may affect data consistency and completeness. The indicators included in this study were developed in practice and reflect practical needs and are regarded as important markers of early development and well-being. The small number of infants with thinness and obesity represents a limitation, as it reduces the precision of the estimates and warrants cautious interpretation of the findings. Other limitations include that the assessments at school entry were based on brief interactions, which may not fully capture the child’s lived experience. The criteria guiding health nurses’ evaluations are not clearly defined, making it unclear under which circumstances they report concerns about a child’s home environment or emotional well-being. These assessments are inherently influenced by the level of trust between the child and the health nurse, which may affect the child’s willingness to disclose sensitive information. As such, the observed development and well-being outcomes may be subject to underreporting or variability in interpretation and could impact the reliability of these development and well-being assessments. Similarly, although official guidelines exist for measuring children’s weight and height, the specific measurement protocol has not been formally validated in this dataset. However, objectively measured height and weight are often considered more reliable than parent-reported measures from questionnaires, which may be subject to recall and reporting bias, particularly for height/length measurements [57].

This study aimed to assess the predictive performance of infant weight status on concerns of later development and well-being without adjustment for additional confounders, as the primary focus was on the individual impact of early weight status. Due to the nature of the research question, external validation of the model was not prioritized, and the full dataset was used for model estimation and performance evaluation. Furthermore, we focused on AUC as the standard measure of overall model performance, providing a threshold-independent assessment of predictive ability. These approaches were deemed appropriate given the exploratory aim and the completeness of the dataset. Using BMI-for-age z-scores allowed weight status to be modeled as a continuous variable, thereby preserving more information than categorical classifications. Nonetheless, this analytical advantage did not translate into improved predictive accuracy. Further research should explore additional or combined factors that better reflect the multifaceted nature to predict child development and well-being.

Although routinely collected data offers cost-effective and ethical advantages, our findings demonstrated that the predictive performance of infant weight status for development and well-being in the first years of school—when based on child records collected as part of the universal offer of Danish health nurses—remained poor. Further research is needed to explore whether this reflects limitations in data or whether it illustrates that infant weight may not capture the complexity of factors contributing to concerns of later development and well-being.

## Supplementary information

Figure S1

Table S1

## Data Availability

The data analyzed in this study are subject to legal and administrative restrictions. Access to the underlying data requires approval from both the relevant Team for Medical Records Research, Capital Region of Denmark, and the individual participating municipalities. Access may be granted upon reasonable request by contacting the corresponding author.
