# Supplementary material for: Can weight status in the first year of life predict well-being in the first years of school?
Source: Int J Obes (Lond). 2026 Aug 20;50(10):2212–21. doi: 10.1038/s41366-026-02180-6 (PMC13722593; doi:10.1038/s41366-026-02180-6)
Supplement: Supplementary file 1 [file 41366_2026_2180_MOESM2_ESM.pdf]

Supplementary

Table S1: Performance of line regression using restricted cubic splines measured in Area Under the Curve (AUC) by stratification of sex and education level

	Stratification of sex		Stratification of education level	
	Boy	Girl	Low education[1]	High education[2]
Concerns about language skills	0.512	0.519	0. 511	0.512
Concerns about motor development	0.517	0.515	0.515	0.520
Concerns about perceived general well-being	0.518	0.510	0.507	0.515
Concerns about perceived school well-being	0.513	0.517	0.516	0.511
Concerns about parent-child relationship	0.509	0.515	0.516	0.517
Concerns based on health nurses' observations on child interaction	0.509	0.509	0.509	0.509
Concerns about peer relationship problems	0.514	0.509	0.515	0.518

[1] Primary and lower secondary education, vocational education and training, general or vocational upper secondary education

[2] 3 years or more at university
